# Supplementary material for: Identification of potential biomarkers in active Lyme borreliosis
Source: PLoS One. 2023 Jun 26;18(6):e0287586. doi: 10.1371/journal.pone.0287586 (PMC10292690; doi:10.1371/journal.pone.0287586)
Supplement: S1 Table — *Highest p-value below 0.1 of two randomly selected halves of the study population calculated using Mann-Whitney’s U-test (for details see the original publication). (DOCX) [file pone.0287586.s001.docx]

**S1 Table.** <LOD; Frequency of samples with results below the lowest concentration possible to detect. *Highest p-value below 0.1 of two randomly selected halves of the study population calculated using Mann-Whitney’s U-test (for details see the original publication)

|  | |  |  |  |  |  |  |  |  |
| --- | --- | --- | --- | --- | --- | --- | --- | --- | --- |
| Median (Q1-Q3) | |  |  |  |  |  |  |  |  |
|  |  |  | **Lyme borreliosis patients** |  | **Blood donors** |  |  |  |  |
| **Protein** | **Uniprot_ID** |  | **n=52** |  | **n=75** |  | **<LOD (%)** |  | **p-value*** |
| **AREG** | **P15514** |  | 2.77 (2.61-3.24) |  | 2.60 (2.37-2.73) |  | 1 |  |  |
| **ARNT** | **P27540** |  | 1.03 (1.03-1.03) |  | 1.03 (1.03-1.03) |  | 89 |  |  |
| **BACH1** | **O14867** |  | 1.44 (1.13-1.75) |  | 1.55 (1.23-2.00) |  | 8 |  |  |
| **BIRC2** | **Q13490** |  | -0.27 (-0.27--0.27) |  | -0.27 (-0.27--0.27) |  | 100 |  |  |
| **BTN3A2** | **P78410** |  | 3.06 (2.83-3.35) |  | 2.94 (2.72-3.23) |  | 1 |  |  |
| **CCL11** | **P51671** |  | 8.35 (8.06-8.59) |  | 8.44 (8.22-8.69) |  | 1 |  |  |
| **CD28** | **P10747** |  | 1.05 (1.05-1.05) |  | 1.05 (1.05-1.05) |  | 99 |  |  |
| **CD83** | **Q01151** |  | 3.16 (2.88-3.38) |  | 3.08 (2.90-3.32) |  | 1 |  |  |
| **CDSN** | **Q15517** |  | 2.51 (2.18-2.88) |  | 2.48 (2.19-2.88) |  | 20 |  |  |
| **CKAP4** | **Q07065** |  | 4.10 (3.96-4.33) |  | 3.93 (3.72-4.15) |  | 1 |  | 0.071 |
| **CLEC4A** | **Q9UMR7** |  | 4.20 (3.96-4.59) |  | 4.23 (4.01-4.40) |  | 1 |  |  |
| **CLEC4C** | **Q8WTT0** |  | 3.97 (3.54-4.60) |  | 4.34 (4.02-4.75) |  | 1 |  |  |
| **CLEC4D** | **Q8WXI8** |  | 3.54 (2.84-4.42) |  | 3.13 (2.69-3.84) |  | 1 |  |  |
| **CLEC4G** | **Q6UXB4** |  | 3.11 (2.87-3.30) |  | 3.02 (2.73-3.17) |  | 1 |  |  |
| **CLEC6A** | **Q6EIG7** |  | 2.88 (2.62-3.23) |  | 2.56 (2.22-3.03) |  | 1 |  |  |
| **CLEC7A** | **Q9BXN2** |  | 2.85 (2.66-3.07) |  | 2.76 (2.46-3.02) |  | 1 |  |  |
| **CNTNAP2** | **Q9UHC6** |  | 1.18 (0.97-1.40) |  | 1.32 (1.05-1.48) |  | 1 |  |  |
| **CXADR** | **P78310** |  | 1.55 (1.27-1.98) |  | 1.57 (1.40-1.91) |  | 1 |  |  |
| **CXCL12** | **P48061** |  | 0.33 (0.33-0.33) |  | 0.33 (0.33-1.39) |  | 73 |  |  |
| **DAPP1** | **Q9UN19** |  | 0.52 (0.52-0.52) |  | 0.52 (0.52-0.52) |  | 89 |  |  |
| **DCBLD2** | **Q96PD2** |  | 8.62 (8.44-8.76) |  | 8.57 (8.35-8.68) |  | 1 |  |  |
| **DCTN1** | **Q14203** |  | 3.45 (2.83-4.09) |  | 3.62 (3.34-4.05) |  | 1 |  |  |
| **DDX58** | **O95786** |  | 2.43 (2.00-2.89) |  | 1.95 (1.75-2.40) |  | 3 |  | 0.037 |
| **DFFA** | **O00273** |  | 4.12 (3.76-4.54) |  | 4.22 (4.06-4.55) |  | 1 |  |  |
| **DGKZ** | **Q13574** |  | 0.06 (0.06-0.06) |  | 0.06 (0.06-0.06) |  | 98 |  |  |
| **DPP10** | **Q8N608** |  | 1.18 (0.96-1.37) |  | 1.18 (1.01-1.35) |  | 1 |  |  |
| **EDAR** | **Q9UNE0** |  | 2.67 (2.10-3.20) |  | 2.59 (2.25-3.09) |  | 8 |  |  |
| **EGLN1** | **Q9GZT9** |  | 1.28 (1.28-2.46) |  | 1.28 (1.28-2.58) |  | 61 |  |  |
| **EIF4G1** | **Q04637** |  | 2.30 (1.84-2.84) |  | 2.59 (2.25-3.03) |  | 1 |  | 0.087 |
| **EIF5A** | **P63241** |  | 0.50 (0.50-0.50) |  | 0.50 (0.50-0.50) |  | 89 |  |  |
| **FAM3B** | **P58499** |  | 4.49 (4.19-4.70) |  | 4.66 (4.38-4.91) |  | 1 |  |  |
| **FCRL3** | **Q96P31** |  | 1.49 (1.10-1.86) |  | 1.49 (1.07-1.80) |  | 18 |  |  |
| **FCRL6** | **Q6DN72** |  | 3.42 (3.06-4.14) |  | 3.51 (3.15-3.77) |  | 1 |  |  |
| **FGF2** | **P09038** |  | -0.54 (-0.54-0.78) |  | -0.54 (-0.54-0.53) |  | 61 |  |  |
| **FXYD5** | **Q96DB9** |  | -0.37 (-0.37-0.92) |  | -0.37 (-0.37-0.73) |  | 67 |  |  |
| **GALNT3** | **Q14435** |  | 0.76 (0.76-0.76) |  | 0.76 (0.76-0.76) |  | 86 |  |  |
| **GLB1** | **P16278** |  | 2.81 (2.00-3.28) |  | 2.42 (2.05-2.82) |  | 1 |  |  |
| **HCLS1** | **P14317** |  | 4.05 (3.45-4.55) |  | 4.17 (3.53-4.79) |  | 2 |  |  |
| **HEXIM1** | **O94992** |  | 3.27 (2.95-3.46) |  | 3.39 (3.10-3.85) |  | 1 |  | 0.052 |
| **HNMT** | **P50135** |  | 8.93 (8.64-9.25) |  | 8.87 (8.56-9.25) |  | 1 |  |  |
| **HSD11B1** | **P28845** |  | 2.73 (2.36-2.98) |  | 2.79 (2.48-3.17) |  | 1 |  |  |
| **ICA1** | **Q05084** |  | 0.98 (0.98-0.98) |  | 0.98 (0.98-0.98) |  | 99 |  |  |
| **IFNLR1** | **Q8IU57** |  | 2.41 (2.24-2.54) |  | 2.47 (2.31-2.63) |  | 1 |  |  |
| **IL10** | **P22301** |  | 3.74 (3.47-4.42) |  | 3.54 (3.31-3.93) |  | 1 |  |  |
| **IL12RB1** | **P42701** |  | 1.85 (0.66-2.08) |  | 1.83 (0.66-2.10) |  | 34 |  |  |
| **IL5** | **P05113** |  | 0.19 (0.19-1.72) |  | 0.19 (0.19-0.19) |  | 73 |  |  |
| **IL6** | **P05231** |  | 3.09 (2.51-3.55) |  | 2.29 (1.87-2.73) |  | 11 |  | 0.004 |
| **IRAK1** | **P51617** |  | 0.85 (0.60-1.22) |  | 0.91 (0.70-1.28) |  | 13 |  |  |
| **IRAK4** | **Q9NWZ3** |  | 1.33 (1.33-1.33) |  | 1.33 (1.33-1.33) |  | 95 |  |  |
| **IRF9** | **Q00978** |  | 1.71 (1.40-1.96) |  | 1.49 (1.27-1.72) |  | 5 |  |  |
| **ITGA11** | **Q9UKX5** |  | 1.70 (1.44-2.05) |  | 1.83 (1.57-2.07) |  | 6 |  |  |
| **ITGA6** | **P23229** |  | 0.75 (0.54-1.00) |  | 0.75 (0.58-1.04) |  | 1 |  |  |
| **ITGB6** | **P18564** |  | 2.66 (2.37-2.98) |  | 3.03 (2.76-3.23) |  | 1 |  | 0.004 |
| **ITM2A** | **O43736** |  | 3.06 (2.67-3.45) |  | 3.75 (3.41-4.03) |  | 1 |  | 0.012 |
| **JUN** | **P05412** |  | -0.55 (-0.55--0.55) |  | -0.55 (-0.55--0.55) |  | 84 |  |  |
| **KLRD1** | **Q13241** |  | 6.78 (6.61-7.22) |  | 6.71 (6.48-7.05) |  | 1 |  |  |
| **KPNA1** | **P52294** |  | 0.58 (0.58-0.58) |  | 0.58 (0.58-0.58) |  | 82 |  |  |
| **KRT19** | **P08727** |  | 2.94 (2.55-3.52) |  | 2.74 (2.31-3.12) |  | 2 |  |  |
| **LAG3** | **P18627** |  | 2.71 (2.37-3.03) |  | 2.74 (2.57-2.93) |  | 1 |  |  |
| **LAMP3** | **Q9UQV4** |  | 3.88 (3.55-4.28) |  | 3.87 (3.40-4.07) |  | 1 |  |  |
| **LILRB4** | **Q8NHJ6** |  | 2.89 (2.53-3.18) |  | 2.71 (2.49-3.01) |  | 1 |  |  |
| **LY75** | **O60449** |  | 2.39 (2.18-2.66) |  | 2.47 (2.31-2.61) |  | 4 |  |  |
| **MASP1** | **P48740** |  | 1.97 (1.72-2.25) |  | 2.00 (1.71-2.18) |  | 2 |  |  |
| **MGMT** | **P16455** |  | 2.06 (1.48-2.75) |  | 2.04 (1.62-2.55) |  | 2 |  |  |
| **MILR1** | **Q7Z6M3** |  | 3.41 (3.05-3.67) |  | 3.30 (3.05-3.69) |  | 1 |  |  |
| **NCR1** | **O76036** |  | 2.21 (1.96-2.38) |  | 2.04 (1.87-2.31) |  | 1 |  |  |
| **NF2** | **P35240** |  | -2.19 (-2.19--2.19) |  | -2.19 (-2.19--2.19) |  | 98 |  |  |
| **NFATC3** | **Q12968** |  | 0.85 (-0.40-1.12) |  | 0.61 (-0.40-1.08) |  | 42 |  |  |
| **NTF4** | **P34130** |  | 0.76 (0.48-1.03) |  | 0.85 (0.63-1.09) |  | 14 |  |  |
| **PADI2** | **Q9Y2J8** |  | -0.05 (-0.05--0.05) |  | -0.05 (-0.05--0.05) |  | 81 |  |  |
| **PIK3AP1** | **Q6ZUJ8** |  | 4.08 (3.46-4.78) |  | 4.17 (3.76-4.81) |  | 1 |  |  |
| **PLXNA4** | **Q9HCM2** |  | 4.60 (4.06-5.22) |  | 4.90 (4.46-5.44) |  | 1 |  |  |
| **PPP1R9B** | **Q96SB3** |  | 2.12 (1.09-2.62) |  | 2.59 (2.29-2.98) |  | 27 |  | 0.076 |
| **PRDX1** | **Q06830** |  | 0.70 (0.50-0.87) |  | 0.69 (0.54-0.80) |  | 12 |  |  |
| **PRDX3** | **P30048** |  | -1.31 (-1.31--1.31) |  | -1.31 (-1.31--1.31) |  | 98 |  |  |
| **PRDX5** | **P30044** |  | 5.57 (4.93-6.17) |  | 4.77 (4.24-5.26) |  | 1 |  | 0.016 |
| **PRKCQ** | **Q04759** |  | -0.40 (-0.40-0.72) |  | -0.40 (-0.40--0.40) |  | 77 |  |  |
| **PSIP1** | **O75475** |  | 2.37 (1.94-2.82) |  | 2.51 (2.12-3.05) |  | 8 |  |  |
| **PTH1R** | **Q03431** |  | 0.87 (0.87-0.87) |  | 0.87 (0.87-1.91) |  | 80 |  |  |
| **SH2B3** | **Q9UQQ2** |  | 0.98 (0.98-2.22) |  | 2.33 (0.98-2.93) |  | 40 |  | 0.008 |
| **SH2D1A** | **O60880** |  | 1.57 (1.33-1.87) |  | 1.52 (1.26-1.75) |  | 5 |  |  |
| **SIT1** | **Q9Y3P8** |  | 1.52 (1.32-1.73) |  | 1.43 (1.16-1.73) |  | 6 |  |  |
| **SPRY2** | **O43597** |  | 1.02 (1.02-1.02) |  | 1.02 (1.02-1.02) |  | 89 |  |  |
| **SRPK2** | **P78362** |  | -0.38 (-0.38-0.67) |  | -0.38 (-0.38-0.91) |  | 62 |  |  |
| **STC1** | **P52823** |  | 6.40 (5.88-6.68) |  | 6.53 (6.19-6.82) |  | 1 |  |  |
| **TANK** | **Q92844** |  | 1.04 (1.04-2.49) |  | 2.09 (1.04-2.60) |  | 48 |  |  |
| **TPSAB1** | **Q15661** |  | 3.81 (3.38-4.21) |  | 4.03 (3.62-4.32) |  | 1 |  |  |
| **TRAF2** | **Q12933** |  | 2.26 (1.94-2.68) |  | 2.37 (2.17-2.73) |  | 4 |  |  |
| **TREM1** | **Q9NP99** |  | 0.49 (0.19-0.84) |  | 0.26 (-0.08-0.55) |  | 1 |  |  |
| **TRIM21** | **P19474** |  | 1.07 (0.73-1.44) |  | 0.95 (0.65-1.14) |  | 11 |  |  |
| **TRIM5** | **Q9C035** |  | 1.30 (-0.02-1.73) |  | 1.19 (-0.02-1.50) |  | 30 |  |  |
| **ZBTB16** | **Q05516** |  | -0.03 (-0.03-1.05) |  | -0.03 (-0.03-1.42) |  | 63 |  |  |
|  |  |  |  |  |  |  |  |  |  |
|  | | | | | | | | | |
|  | | | | | | | | | |
|  | | | | | |  |  |  |  |
