# Supplementary material for: Identification of potential biomarkers in active Lyme borreliosis
Source: PLoS One. 2023 Jun 26;18(6):e0287586. doi: 10.1371/journal.pone.0287586 (PMC10292690; doi:10.1371/journal.pone.0287586)
Supplement: Attachment — Submitted filename: PONE-D-23-05893_reviewer.pdf [file pone.0287586.s002.pdf]

PLOS ONE

A novel laboratory approach to discriminate active Lyme borreliosis from previous Borrelia exposure

--Manuscript Draft--

**Manuscript Number:**	PONE-D-23-05893
**Article Type:**	Research Article
**Full Title:**	A novel laboratory approach to discriminate active Lyme borreliosis from previous Borrelia exposure
**Short Title:**	A novel laboratory approach to discriminate active Lyme borreliosis from previous Borrelia exposure
**Corresponding Author:**	Ivar Tjernberg, M.D., Ph.D., Assoc prof<br>Linköping University: Linkopings universitet<br>Kalmar, SWEDEN
**Keywords:**	Lyme borreliosis, laboratory diagnosis, proteomics, ongoing infection, serology
**Abstract:**	Objectives<br>Lyme serology does not readily discriminate an ongoing Lyme borreliosis (LB) infection from a previous exposure. Here, we aimed to investigate a large number of immunological protein biomarkers to search for an immunological pattern typical for active LB, in contrast to patterns found in individuals previously exposed for Borrelia and healthy blood donors.<br>Methods<br>Serum samples from well-characterised adult patients with ongoing LB and healthy blood donors were included and investigated using a proximity extension assay (provided by Olink®) by which 92 different immune response-related human protein biomarkers were analysed simultaneously.<br>Results<br>In total, 52 LB patients and 75 healthy blood donors were included. The blood donors represented both previously Borrelia exposed (n=34) and not exposed (n=41) based on anti-Borrelia antibody status. Ten of the examined 92 immune-related proteins differed between patients and blood donors and were chosen for further logistic regression (p<0.1). Six proteins were statistically significant different between LB patients and blood donors (p<0.05); PPP1R9B, PRDX5, ITM2A, EIF4GI, DDX58, and ITGB6. These six proteins were then combined in an index and analysed using receiver-operating-characteristic curve analysis showing an area under the curve of 0.964 (p<0.001).<br>Conclusions<br>The results from this study suggest that there is an immunological protein pattern that can distinguish a present Borrelia infection from a previous exposure as well as anti-Borrelia antibody negative blood donors. Although this method is not adapted for routine clinical use at this point, the possibility is interesting and may open new diagnostic opportunities improving the laboratory diagnostics of LB.
**Order of Authors:**	Ivar Tjernberg, M.D., Ph.D., Assoc prof
	Malin Lager, PhD
	Guro Furset Jensen, MD
	Randi Eikeland, MD, Assoc prof
	Dag Nyman, MD, professor
	Lars Brudin, MD, professor
	Anna J Henningsson, MD, Senior assoc. prof
**Additional Information:**	
**Question**	**Response**
**Financial Disclosure**	This study was supported by university grants to Region Kalmar County (IT), Futurum-

Enter a financial disclosure statement that describes the sources of funding for the work included in this submission. Review the submission guidelines for detailed requirements. View published research articles from *PLOS ONE* for specific examples.<br><br>This statement is required for submission and **will appear in the published article** if the submission is accepted. Please make sure it is accurate.<br><br>**Unfunded studies**<br>Enter: *The author(s) received no specific funding for this work.*<br><br>**Funded studies**<br>Enter a statement with the following details:<br>• Initials of the authors who received each award<br>• Grant numbers awarded to each author<br>• The full name of each funder<br>• URL of each funder website<br>• Did the sponsors or funders play any role in the study design, data collection and analysis, decision to publish, or preparation of the manuscript?<br>• **NO** - Include this sentence at the end of your statement: *The funders had no role in study design, data collection and analysis, decision to publish, or preparation of the manuscript.*<br>• **YES** - Specify the role(s) played.<br><br>* typeset	Academy for Healthcare, Region Jönköping County, Division of Medical Diagnostics, Region of<br>Jönköping County, Interreg IVA Program ScandTick (grant no. 167226) and Interreg V program ScandTick Innovation (project ID. 20200422, reference no. 2015-29 000167), The Medical Research Council of Southeast Sweden (FORSS-475511), the foundation for medical research of the Åland cultural foundation and the Wilhelm and Else Stockmann Foundation (AJH). The funders had no role in study design, data collection and analysis, decision to publish, or preparation of the manuscript.
**Competing Interests**<br><br>Use the instructions below to enter a competing interest statement for this submission. On behalf of all authors, disclose any competing interests that could be perceived to bias this work—acknowledging all financial support and any other relevant financial or non-financial competing interests.<br><br>This statement is required for submission and **will appear in the published article** if	I have read the journal's policy and the authors of this manuscript have the following competing interests: IT reports participation in advisory board and personal fees from Pfizer Inc outside the submitted work. AJH has a collaborative research agreement with Abbott Laboratories, Chicago, USA and Reagena Oy, Toivala, Finland. Remaining authors report no conflict of interest.

the submission is accepted. Please make sure it is accurate and that any funding sources listed in your Funding Information later in the submission form are also declared in your Financial Disclosure statement.<br><br>View published research articles from *PLOS ONE* for specific examples.<br><br>**NO authors have competing interests**<br><br>Enter: *The authors have declared that no competing interests exist.*<br><br>**Authors with competing interests**<br><br>Enter competing interest details beginning with this statement:<br><br>*I have read the journal's policy and the authors of this manuscript have the following competing interests: [insert competing interests here]*<br><br>* typeset	
**Ethics Statement**<br><br>Enter an ethics statement for this submission. This statement is required if the study involved:<br><br>• Human participants<br>• Human specimens or tissue<br>• Vertebrate animals or cephalopods<br>• Vertebrate embryos or tissues<br>• Field research<br><br>Write "N/A" if the submission does not require an ethics statement.<br><br>General guidance is provided below. Consult the submission guidelines for detailed instructions. **Make sure that all information entered here is included in the Methods section of the manuscript.**	The study was approved by the respective regional ethical review boards in Sweden, Åland and Norway. For Sweden 2013/238-31, 2014/326-32, 2016/211-31, 2019-03832, for Åland the approval was given at the regional ethical review board meeting no 2/2014 and for Norway 2014/1100 and additionally September 13, 2019 with reference no 9463. All study participants agreed to take part in the study and signed a written informed consent form.

**Format for specific study types**<br><br>**Human Subject Research (involving human participants and/or tissue)**<br>• Give the name of the institutional review board or ethics committee that approved the study<br>• Include the approval number and/or a statement indicating approval of this research<br>• Indicate the form of consent obtained (written/oral) or the reason that consent was not obtained (e.g. the data were analyzed anonymously)<br><br>**Animal Research (involving vertebrate animals, embryos or tissues)**<br>• Provide the name of the Institutional Animal Care and Use Committee (IACUC) or other relevant ethics board that reviewed the study protocol, and indicate whether they approved this research or granted a formal waiver of ethical approval<br>• Include an approval number if one was obtained<br>• If the study involved *non-human primates*, add *additional details* about animal welfare and steps taken to ameliorate suffering<br>• If anesthesia, euthanasia, or any kind of animal sacrifice is part of the study, include briefly which substances and/or methods were applied<br><br>**Field Research**<br><br>Include the following details if this study involves the collection of plant, animal, or other materials from a natural setting:<br>• Field permit number<br>• Name of the institution or relevant body that granted permission	
**Data Availability**<br><br>Authors are required to make all data underlying the findings described fully available, without restriction, and from the time of publication. PLOS allows rare exceptions to address legal and ethical concerns. See the PLOS Data Policy and FAQ for detailed information.	No - some restrictions will apply

A Data Availability Statement describing where the data can be found is required at submission. Your answers to this question constitute the Data Availability Statement and **will be published in the article**, if accepted.<br><br>**Important:** Stating 'data available on request from the author' is not sufficient. If your data are only available upon request, select 'No' for the first question and explain your exceptional situation in the text box.<br><br>Do the authors confirm that all data underlying the findings described in their manuscript are fully available without restriction?	
**Describe where the data may be found in full sentences. If you are copying our sample text, replace any instances of XXX with the appropriate details.**<br><br>• If the data are **held or will be held in a public repository**, include URLs, accession numbers or DOIs. If this information will only be available after acceptance, indicate this by ticking the box below. For example: *All XXX files are available from the XXX database (accession number(s) XXX, XXX.*).<br>• If the data are all contained **within the manuscript and/or Supporting Information files**, enter the following: *All relevant data are within the manuscript and its Supporting Information files.*<br>• If neither of these applies but you are able to provide **details of access elsewhere**, with or without limitations, please do so. For example:<br><br>*Data cannot be shared publicly because of [XXX]. Data are available from the XXX Institutional Data Access / Ethics Committee (contact via XXX) for researchers who meet the criteria for access to confidential data.*<br><br>*The data underlying the results presented in the study are available from (include the name of the third party*	Data cannot be shared publicly due to ethical study decisions in ethical review boards. Data are available from Linköping University, Sweden for researchers who meet the criteria for access to confidential data.

and contact information or URL).<br>• This text is appropriate if the data are owned by a third party and authors do not have permission to share the data.<br><br>* typeset	
Additional data availability information:	

Powered by Editorial Manager® and ProduXion Manager® from Aries Systems Corporation

Manuscript

Click here to access/download;Manuscript;Manuscript Active Lyme borreliosis_R1.docx

A novel laboratory approach to discriminate active Lyme borreliosis from previous *Borrelia* exposure

Ivar Tjernberg*, Malin Lager, Guro Furset Jensen, Randi Eikeland, Dag Nyman, Lars Brudin, Anna J. Henningsson

*Corresponding author

Ivar Tjernberg, MD, assoc. prof

Department of Clinical Chemistry and Transfusion Medicine, Region Kalmar County,

and Department of Biomedical and Clinical Sciences, Division of Inflammation and Infection, Linköping University, Linköping, Sweden

ORCID: 0000-0001-8657-2496

E-mail: ivar.tjernberg@liu.se

Telephone: +46 76 1184486

Malin Lager, PhD

National Reference Laboratory for Borrelia, Department of Clinical Microbiology in Jönköping, Region Jönköping County, Linköping University, Linköping, Sweden

Guro Furset Jensen, MD

a) National Reference Laboratory for Borrelia, Department of Clinical Microbiology, Sørlandet Hospital Trust, Kristiansand, Norway

b) Department of Clinical Microbiology, Sørlandet Hospital Health Enterprise, Kristiansand, Norway

Randi Eikeland, MD, assoc. prof

a) National Advisory unit on Tick borne Diseases, Sørlandet Hospital Trust, Kristiansand, Norway

b) Faculty of health and sports science, University of Agder, Grimstad, Norway

c) ESCMID Study Group for Lyme Borreliosis—ESGBOR, part of the European Society for Clinical Microbiology and Infectious Diseases, Basel, Switzerland

Dag Nyman, MD, professor

a) The Åland group for borrelia research

b) ESCMID Study Group for Lyme Borreliosis—ESGBOR, part of the European Society for Clinical Microbiology and Infectious Diseases, Basel, Switzerland

Lars Brudin, MD, professor

Department of Clinical Physiology, Kalmar, Region Kalmar County and Department of Health, Medicine and Caring Sciences, Linköping University, Linköping, Sweden

Anna J Henningsson, MD, Senior assoc. prof

a) National Reference Laboratory for Borrelia and Other Tick-Borne Bacteria, Department of Clinical Microbiology in Jönköping, Region Jönköping County, Linköping University, Linköping, Sweden

b) Department of Biomedical and Clinical Sciences, Linköping University, Linköping, Sweden

c) ESCMID Study Group for Lyme Borreliosis—ESGBOR, part of the European Society for Clinical Microbiology and Infectious Diseases, Basel, Switzerland

Abstract

Objectives

Lyme serology does not readily discriminate an ongoing Lyme borreliosis (LB) infection from a previous exposure. Here, we aimed to investigate a large number of immunological protein biomarkers to search for an immunological pattern typical for active LB, in contrast to patterns found in individuals previously exposed for *Borrelia* and healthy blood donors.

Methods

Serum samples from well-characterised adult patients with ongoing LB and healthy blood donors were included and investigated using a proximity extension assay (provided by Olink®) by which 92 different immune response-related human protein biomarkers were analysed simultaneously.

Results

In total, 52 LB patients and 75 healthy blood donors were included. The blood donors represented both previously *Borrelia* exposed ($n=34$) and not exposed ($n=41$) based on anti-*Borrelia* antibody status. Ten of the examined 92 immune-related proteins differed between patients and blood donors and were chosen for further logistic regression ($p<0.1$). Six proteins were statistically significant different between LB patients and blood donors ($p<0.05$); PPP1R9B, PRDX5, ITM2A, EIF4GI, DDX58, and ITGB6. These six proteins were then combined in an index and analysed using receiver-operating-characteristic curve analysis showing an area under the curve of 0.964 ($p<0.001$).

Conclusions

The results from this study suggest that there is an immunological protein pattern that can distinguish a present *Borrelia* infection from a previous exposure as well as anti-*Borrelia*

antibody negative blood donors. Although this method is not adapted for routine clinical use at this point, the possibility is interesting and may open new diagnostic opportunities improving the laboratory diagnostics of LB.

Introduction

Lyme borreliosis (LB) is considered the most common tick-borne disease in both Europe and North America [1-3]. The disease, caused by bacteria belonging to the *Borrelia burgdorferi* sensu lato (s.l.) complex, may give rise to a number of different clinical manifestations including erythema migrans, Lyme neuroborreliosis, acrodermatitis chronica atrophicans, Lyme arthritis, and other more rare manifestations [2]. However, *Borrelia* exposure may also pass sub-clinically in a significant proportion of exposed individuals [3-5]. In the routine clinical setting, diagnosis of erythema migrans relies on patient history and physical examination, whereas in the remaining manifestations laboratory support is also required in the diagnostic workup. Although much effort has been invested in the development of direct detection by molecular methods, these are not widely used due to low sensitivity in some materials, e.g. cerebrospinal fluid. In clinical practice, indirect methods, essentially antibody detection by serology, remains the gold standard for the laboratory diagnosis of LB [2, 6]. However, there are challenges in the interpretation of anti-*Borrelia* antibody results that must be considered in the diagnosis of LB. The serological results must be interpreted in relation to the variable clinical presentation of LB including the dissemination of the infection. It is important to recognise that a single positive serological result does not discriminate an active infection from a previous symptomatic or subclinical exposure [6, 7]. Furthermore, the local epidemiological situation needs to be considered. In highly endemic areas, the seroprevalence may reach high figures in the healthy population thus significantly reducing the positive predictive value of a positive test result [8, 9]. In addition, test cross-reactions may also render false positive serological results [6, 10, 11]. However, the most important limitation of *Borrelia* serology in the routine clinical setting in endemic areas is that the population is highly seropositive after previous exposure to *Borrelia*. Additional tests to discriminate an active from a previous LB infection would improve the diagnostic process considerably and

limit the risk for overdiagnosing and thereby counteract overuse of antibiotics with associated risks for side-effects for patients as well as the negative effects on the environment including antibiotic resistance. In this study, we wanted to investigate serum samples from clinically well characterised LB patients and healthy blood donors examining a large number of immunological protein biomarkers in order to possibly distinguish active LB from previous *Borrelia* exposure.

Material and methods

Lyme borreliosis patients and blood donors

A subset of serum samples from a previous prospective study of well characterised adult LB patients together with serum samples from healthy blood donors originally included during 2013-2017 were included in this study [12]. Both blood donors with and without anti-*Borrelia* antibodies were included in order to also cover individuals with a previous exposure to *B. burgdorferi* s.l. In total, 52 samples were available from patients with different manifestations of LB, Table 1. Serum samples were obtained from the patients before treatment initiation. Classification and confirmation of LB diagnosis in the patients were based on clinical information including laboratory results and review of medical records by experienced physicians in the field. For comparison, samples from 80 blood donors screened for anti-*Borrelia* antibodies using Enzygnost Borrelia Lyme IgM/IgG (Siemens/DADE Behring, Marburg, Germany) were investigated. These 80 blood donors comprised 37 IgM and/or IgG anti-*Borrelia* antibody positive (34 IgG-positive and three IgM and IgG-positive) and 43 anti-*Borrelia* antibody negative subjects. Blood donors were only included after an approved health declaration and no current symptoms or signs of disease. After classification of LB patients and blood donors data was anonymised. Further details and definitions of the study subjects have been described in a previous publication [12].

Table 1

Demographic characteristics of patients and blood donors (n=132)

**Clinical group**	**Lyme borreliosis patients (n=52)**			**Blood donors (n=80)**	
	**Definite LNB (n=41)**	**ACA/LA (n=7)**	**Erythema migrans (n=4)**	**Blood donors AB+ (n=37)**	**Blood donors AB− (n=43)**
Sex, female/male (% female)	25/16 (70)	4/3 (57)	3/1 (75)	7/30 (19)	14/29 (33)
Median age in years (range)	61 (21-85)	48 (27-55)	51 (45-69)	47 (20-68)	48 (21-66)

LNB, Lyme neuroborreliosis; ACA, Acrodermatitis chronica atrophicans; LA, Lyme arthritis;
AB+, anti-*Borrelia* antibody positive; AB−, anti-*Borrelia* antibody negative

Laboratory analyses

Serum samples were all investigated using a proximity extension assay provided by the Clinical Biomarkers facility, Science for Life Laboratory, Uppsala University, Sweden. In short, multiple analytes are detected in parallel by individually paired matched antibodies labelled with unique strands of DNA oligonucleotides that may hybridize when in proximity with each other, allowing for subsequent amplification, e.g. using quantitative PCR, in which the results are related to the initial concentration of each target. The panel used was the immune response panel v.3202 covering 92 immune response-related human protein biomarkers. A comprehensive list of analytes covered in the panel may be found at the Olink website [13]. Proteins in this publication have been referenced by their respective gene as well as their unique protein identification number (UniProt ID) according to the Universal Protein Resource (www.uniprot.org).

Data analysis and statistics

Results for all parameters were presented as normalised protein expression values on a logarithmic scale with base 2 ($\log_2$) based on the respective analytical cycle threshold values.

The lowest concentration possible to detect (LOD) for each of the proteins is given. Individual values below LOD are replaced by LOD-1 which represents 50% of LOD. LOD and number of values below LOD (%) per protein are presented in the supplementary Table 1. Parameters with at least 50% values within the detectable range were chosen for the subsequent analysis. The further analyses were as follows: 1) The database was randomly divided in two halves, equally for both LB patients and blood donors, and the difference between the two groups within each half analysed separately with Mann-Whitney's U-test. This was done in order to reduce random statistical effects. 2) Secondly, the differences between LB patients and blood donors, with a significance level of <0.1 simultaneously for

the two groups were qualified for successive analyses with multivariate logistic regression with $p<0.05$ as a criterion. These were then subjected to form an index using a linear equation with the log(OR) as weights for the various proteins, then presented in receiver-operating characteristic (ROC) analysis. Statistical analyses were performed in Statistica version 13.

Ethics

The study was approved by the respective regional ethical review boards in Sweden, Åland and Norway. For Sweden 2013/238-31, 2014/326-32, 2016/211-31, 2019-03832, for Åland the approval was given at the regional ethical review board meeting no 2/2014 and for Norway 2014/1100 and additionally September 13, 2019 with reference no 9463. All study participants agreed to take part in the study and signed a written informed consent form.

Results

Basic demographics of the LB patients and the blood donors are shown in Table 1. The LB patients (n=52) consisted of the following manifestations; Lyme neuroborreliosis (n=41), acrodermatitis chronica atrophicans and/or Lyme arthritis (n=7) and erythema migrans (n=4). Further details regarding the subjects may be found in the publication by Lager *et al* [12]. Thus, LB patients were all sampled during an ongoing manifestation of LB, while Results from five subjects in the blood donor group (three anti-*Borrelia* antibody positive and two anti-*Borrelia* antibody negative) were not accepted in the quality control in the proximity extension assay and were therefore excluded from further analyses. Median normalised protein expression values in $\log_2$ scale together with quartile one-three results for the included 92 parameters from the remaining 52 LB patients and 75 blood donors (34 anti-*Borrelia* antibody positive and 41 anti-*Borrelia* antibody negative) are shown in supplementary Table 1. In total, ten of the 92 variables showed a difference with $p<0.1$, further included in a logistic regression model in which six (PPP1R9B, PRDX5, ITM2A, EIF4GI, DDX58, ITGB6) of the ten protein markers remained ($p<0.05$), Table 2. Furthermore, an index was created using the individual results for these six protein markers:

$$\text{Index} = \text{PPP1R9B}*(-1.962)+\text{PRDX5}*(2.307)+\text{ITM2A}*(-2.632)+\text{EIF4G1}*(-2.477)+\text{DDX58}*(2.295)+\text{ITGB6}*(-2.121)$$

Table 2

Median (Q1-Q3) of the ten proteins qualified for the successive multivariate logistic regression. OR is odds ratio with 95% confidence limits where control is reference.

Protein	Uniprot_ID	Lyme borreliosis patients n=52	Blood donors n=75	p-value*	Odds ratio (95% CL)	p-value
CKAP4	Q07065	4.10 (3.96-4.33)	3.93 (3.72-4.15)	0.071		
DDX58	O95786	2.43 (2.00-2.89)	1.95 (1.75-2.40)	0.037	9.93 (1.49-66.21)	0.018
EIF4G1	Q04637	2.30 (1.84-2.84)	2.59 (2.25-3.03)	0.087	0.08 (0.01-0.58)	0.013
HEXIM1	O94992	3.27 (2.95-3.46)	3.39 (3.10-3.85)	0.052		
IL6	P05231	3.09 (2.51-3.55)	2.29 (1.87-2.73)	0.004		
ITGB6	P18564	2.66 (2.37-2.98)	3.03 (2.76-3.23)	0.004	0.12 (0.01-0.97)	0.046
ITM2A	O43736	3.06 (2.67-3.45)	3.75 (3.41-4.03)	0.012	0.07 (0.01-0.41)	0.003
PPP1R9B	Q96SB3	2.12 (1.09-2.62)	2.59 (2.29-2.98)	0.076	0.14 (0.04-0.51)	0.003
PRDX5	P30044	5.57 (4.93-6.17)	4.77 (4.24-5.26)	0.016	10.0 (2.0-50.6)	0.006
SH2B3	Q9UQQ2	0.98 (0.98-2.22)	2.33 (0.98-2.93)	0.008		

*) p-values from Mann-Whitneys U-test (<0.1), necessary for inclusion into the succeeding logistic regression (for details see "Methods").

For comparison between LB patients and blood donors, this index was used in a subsequent ROC curve analysis shown in Fig 1. A significant difference ($p<0.001$) was shown with an area under curve of 0.964. Using an index criterion value of >−8.8178 to discriminate LB patients from blood donors, a sensitivity of 86% and a specificity of 92% could be achieved. As shown by the index calculation, high values of PRDX5 and DDX58 in combination with low values for the remaining markers were typical for the LB patients, while the opposite pattern was characteristic for the blood donors. Age and sex had no impact on the odds ratio of the index (p-values for odds ratios were for age 0.68 and for sex 0.73; data not shown in Table).

Fig 1. Receiver operating characteristic curve displaying the diagnostic power in the six protein index discriminating Lyme borreliosis patients from blood donors. AUC, area under curve.

Discussion

We here show the possibility to successfully distinguish active ongoing LB from blood donors with and without previous exposure to *Borrelia* using a multiplex biochemical approach independent from *Borrelia* serology. The results in this study imply that a number of immune-related serum protein biomarkers may serve this purpose.

In detail, the results show that six proteins are indicated, namely the gene products corresponding to PPP1R9B, PRDX5, ITM2A, EIF4GI, DDX58, and ITGB6. These proteins serve different functions in the human body briefly described here. PPP1R9B, protein phosphatase 1 regulatory subunit 9B, also named neurabin2 and spinophilin seems to be involved in multiple signalling pathways as a scaffold protein. Furthermore, it has been reported to play a significant role as a link between actin cytoskeleton and the plasma membrane at synaptic junctions as well as a G-protein signalling regulator. It also appears that this protein is of special importance in the nervous system [14, 15]. PRDX5, peroxiredoxin-5, also known as PMP20 is a thiol-specific peroxidase that catalyses reduction of organic hydroperoxides and hydrogen peroxide thereby playing a role in cell protection against oxidative stress [15, 16]. ITM2A, integral membrane protein 2A, has been reported to play a role in regulating the development and function of T cells, but additional data on the function of this protein is lacking [17]. EIF4GI, eukaryotic translation initiation factor 4 gamma 1, constitutes a part of the protein complex eIF4F, central for ribosome protein translation initiation and its function thereby regulates protein synthesis. Several viruses have been reported to modify the function of EIF4G by proteolytic cleavage resulting in down-regulation of cellular translation initiation [18], thus a target protein that may be relevant for host defence in infections. DDX58 corresponds to an antiviral innate immune response receptor retinoic acid-induced gene-I (RIG-I), that senses viral nucleic acids in the cytoplasm

and further activates a signalling cascade that leads to the production of pro-inflammatory cytokines and type I interferons, which also may play important roles in bacterial infections [19]. Finally, ITGB6, integrin subunit beta 6, constitutes a part of the heterodimeric integrin $\alpha v\beta 6$ transmembrane receptor exclusively expressed in epithelial cells. Integrin $\alpha v\beta 6$ may serve as a receptor for fibronectin, but one of its major functions is to activate transforming growth factor-$\beta 1$, a key regulator of the innate anti-inflammatory surveillance of the body [20]. Taken together, the combined profile suggests that these six proteins may represent relevant reactions caused by the pathogen in combination with the host immune defence thus including cellular signal transduction, regulation of oxidative stress and protein synthesis, and regulation of cellular immunity as well as inflammatory and anti-inflammatory reactions necessary for clearance of the pathogen. Interestingly, the markers may be both up- and downregulated, and in ongoing LB both PRDX5 and DDX58 were upregulated while the remaining markers were downregulated, suggesting importance of this specific pattern in the present ongoing LB infection phase. To our knowledge, these six markers have not previously been investigated and shown relevant in the perspective of LB. Thus, the significance of them in the discrimination of active from previous LB needs further confirmation in other clinical LB materials as well as in differential conditions to LB. Furthermore, longitudinal studies of confirmed LB patients with follow-up sampling after treatment and resolution of symptoms would be of great value as well as patients classified after additional and other LB serological assays.

The strengths of this study include the well-characterised and relevant clinical LB patients together with subjects representing both healthy controls (blood donors) with and without anti-*Borrelia* antibodies. Moreover, the included LB patients represent the major and most important of the various clinical manifestations of LB in Europe. Additionally, the proximity extension assay methodology applied to the serum material is sensitive and specific [21].

Regarding limitations, although a reasonable number of LB patients and blood donors were investigated, the material is still limited in number, and follow-up samples were not available for the patients which would have been a valuable source of confirmation. Also, the number of studied parameters in relation to the number of available samples needs to be handled with caution in order to avoid random statistical effects.

To conclude, we here show a possibility to discriminate ongoing LB from previous exposure to *Borrelia* using a powerful multiplex serum protein approach. This new strategy is interesting and opens for improvements in the diagnostics of LB in principle although not adapted for clinical use at this point. Further studies on well-characterised clinical materials including follow-up samples and relevant control conditions are warranted. Additional screening using multiplex technology may confirm our data and possibly also detect new and fewer biomarkers with the same diagnostic power suitable for routine use.

Acknowledgements

The authors would like to acknowledge support of the Clinical biomarker facility at SciLifeLab Sweden for providing assistance in protein analyses. We would also like to thank the patients and blood donors for participating in this study as well as all involved health care professionals and departments for valuable assistance.

References

1. Kugeler KJ, Schwartz AM, Delorey MJ, Mead PS, Hinckley AF. Estimating the Frequency of Lyme Disease Diagnoses, United States, 2010-2018. Emerg Infect Dis. 2021;27(2):616-9. Epub 2021/01/27. doi: 10.3201/eid2702.202731. PubMed PMID: 33496229; PubMed Central PMCID: PMCPMC7853543.
2. Stanek G, Strle F. Lyme borreliosis-from tick bite to diagnosis and treatment. FEMS Microbiol Rev. 2018;42(3):233-58. Epub 2018/06/13. doi: 10.1093/femsre/fux047. PubMed PMID: 29893904.
3. Steere AC, Strle F, Wormser GP, Hu LT, Branda JA, Hovius JW, et al. Lyme borreliosis. Nat Rev Dis Primers. 2016;2:16090. Epub 2016/12/16. doi: 10.1038/nrdp.2016.90. PubMed PMID: 27976670; PubMed Central PMCID: PMCPMC5539539.
4. Carlsson H, Ekerfelt C, Henningsson AJ, Brudin L, Tjernberg I. Subclinical Lyme borreliosis is common in south-eastern Sweden and may be distinguished from Lyme neuroborreliosis by sex, age and specific immune marker patterns. Ticks Tick Borne Dis. 2018;9(3):742-8. Epub 2018/03/06. doi: 10.1016/j.ttbdis.2018.02.011. PubMed PMID: 29502989.
5. Wilhelmsson P, Fryland L, Lindblom P, Sjowall J, Ahlm C, Berglund J, et al. A prospective study on the incidence of Borrelia burgdorferi sensu lato infection after a tick bite in Sweden and on the Aland Islands, Finland (2008-2009). Ticks Tick Borne Dis. 2016;7(1):71-9. doi: 10.1016/j.ttbdis.2015.08.009. PubMed PMID: 26341726.
6. Lohr B, Fingerle V, Norris DE, Hunfeld KP. Laboratory diagnosis of Lyme borreliosis: Current state of the art and future perspectives. Crit Rev Clin Lab Sci. 2018;55(4):219-45. Epub 2018/04/03. doi: 10.1080/10408363.2018.1450353. PubMed PMID: 29606016.
7. Dessau RB, van Dam AP, Fingerle V, Gray J, Hovius JW, Hunfeld KP, et al. To test or not to test? Laboratory support for the diagnosis of Lyme borreliosis: a position paper of ESGBOR, the ESCMID study group for Lyme borreliosis. Clin Microbiol Infect. 2018;24(2):118-24. Epub 2017/09/10. doi: 10.1016/j.cmi.2017.08.025. PubMed PMID: 28887186.
8. Carlsson SA, Granlund H, Nyman D, Wahlberg P. IgG seroprevalence of Lyme borreliosis in the population of the Aland Islands in Finland. Scand J Infect Dis. 1998;30(5):501-3. Epub 1999/03/05. doi: 10.1080/00365549850161520. PubMed PMID: 10066053.
9. Johansson M, Manfredsson L, Wistedt A, Serrander L, Tjernberg I. Significant variations in the seroprevalence of C6 ELISA antibodies in a highly endemic area for Lyme borreliosis: evaluation of age, sex and seasonal differences. APMIS. 2017;125(5):476-81. Epub 2017/02/23. doi: 10.1111/apm.12664. PubMed PMID: 28225145.
10. Eldin C, Parola P, Raoult D. Limitations of diagnostic tests for bacterial infections. Med Mal Infect. 2019;49(2):98-101. Epub 2019/01/29. doi: 10.1016/j.medmal.2018.12.004. PubMed PMID: 30686500.
11. Tjernberg I, Kruger G, Eliasson I. C6 peptide ELISA test in the serodiagnosis of Lyme borreliosis in Sweden. Eur J Clin Microbiol Infect Dis. 2007;26(1):37-42. Epub 2006/12/21. doi: 10.1007/s10096-006-0239-3. PubMed PMID: 17180348.
12. Lager M, Dessau RB, Wilhelmsson P, Nyman D, Jensen GF, Matussek A, et al. Serological diagnostics of Lyme borreliosis: comparison of assays in twelve clinical laboratories in Northern Europe. Eur J Clin Microbiol Infect Dis. 2019;38(10):1933-45. Epub 2019/08/11. doi: 10.1007/s10096-019-03631-x. PubMed PMID: 31399914; PubMed Central PMCID: PMCPMC6778534.
13. Olink. Immune response panel Uppsala, Sweden2021. Available from: https://www.olink.com/content/uploads/2021/09/1051-v1.2-immune-response-panel-content-final.pdf.
14. Sarrouilhe D, di Tommaso A, Metaye T, Ladeveze V. Spinophilin: from partners to functions. Biochimie. 2006;88(9):1099-113. Epub 20060517. doi: 10.1016/j.biochi.2006.04.010. PubMed PMID: 16737766.

15. UniProt Consortium T. UniProt: the universal protein knowledgebase. Nucleic Acids Res. 2018;46(5):2699. doi: 10.1093/nar/gky092. PubMed PMID: 29425356; PubMed Central PMCID: PMCPMC5861450.

16. Yamashita H, Avraham S, Jiang S, London R, Van Veldhoven PP, Subramani S, et al. Characterization of human and murine PMP20 peroxisomal proteins that exhibit antioxidant activity in vitro. J Biol Chem. 1999;274(42):29897-904. doi: 10.1074/jbc.274.42.29897. PubMed PMID: 10514471.

17. Tai TS, Pai SY, Ho IC. Itm2a, a target gene of GATA-3, plays a minimal role in regulating the development and function of T cells. PLoS One. 2014;9(5):e96535. Epub 20140515. doi: 10.1371/journal.pone.0096535. PubMed PMID: 24831988; PubMed Central PMCID: PMCPMC4022677.

18. Byrd MP, Zamora M, Lloyd RE. Translation of eukaryotic translation initiation factor 4GI (eIF4GI) proceeds from multiple mRNAs containing a novel cap-dependent internal ribosome entry site (IRES) that is active during poliovirus infection. J Biol Chem. 2005;280(19):18610-22. Epub 20050308. doi: 10.1074/jbc.M414014200. PubMed PMID: 15755734.

19. Rehwinkel J, Gack MU. RIG-I-like receptors: their regulation and roles in RNA sensing. Nat Rev Immunol. 2020;20(9):537-51. Epub 20200313. doi: 10.1038/s41577-020-0288-3. PubMed PMID: 32203325; PubMed Central PMCID: PMCPMC7094958.

20. Koivisto L, Bi J, Hakkinen L, Larjava H. Integrin alphavbeta6: Structure, function and role in health and disease. Int J Biochem Cell Biol. 2018;99:186-96. Epub 20180418. doi: 10.1016/j.biocel.2018.04.013. PubMed PMID: 29678785.

21. Assarsson E, Lundberg M, Holmquist G, Bjorkesten J, Thorsen SB, Ekman D, et al. Homogenous 96-plex PEA immunoassay exhibiting high sensitivity, specificity, and excellent scalability. PLoS One. 2014;9(4):e95192. Epub 20140422. doi: 10.1371/journal.pone.0095192. PubMed PMID: 24755770; PubMed Central PMCID: PMCPMC3995906.

Supporting information

S1 Table.

 Click here to access/download;Figure;Fig1.docx

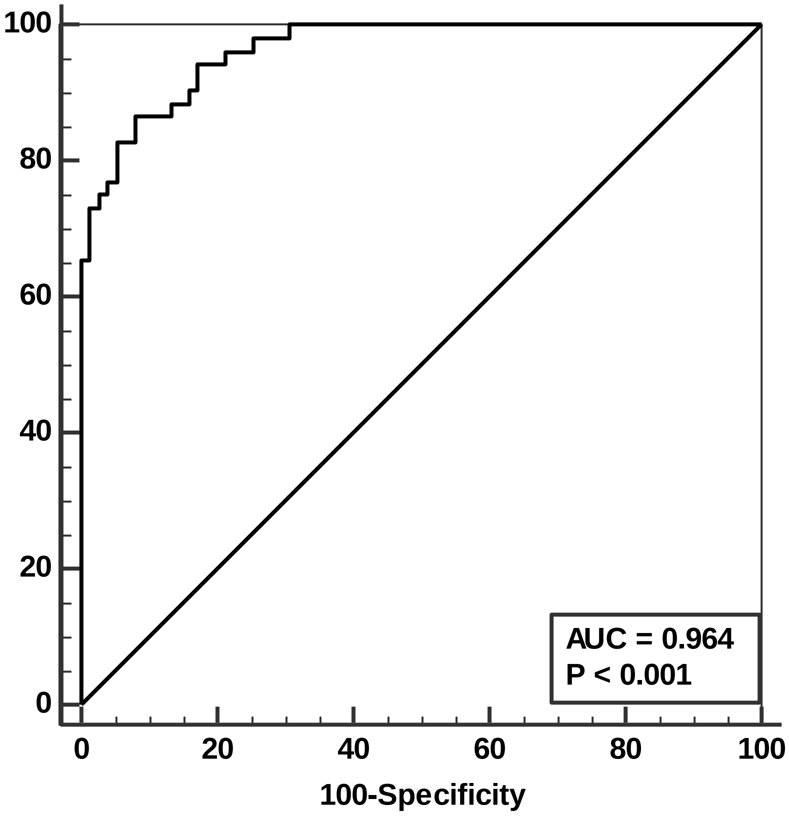

Click here to access/download
Supporting Information
S1_Table.docx
