# Supplementary material for: Identification of potential biomarkers in active Lyme borreliosis
Source: PLoS One. 2023 Jun 26;18(6):e0287586. doi: 10.1371/journal.pone.0287586 (PMC10292690; doi:10.1371/journal.pone.0287586)
Supplement: Attachment — Submitted filename: Responses to reviewers.docx [file pone.0287586.s003.docx]

PONE-D-23-05893 with title: “A novel laboratory approach to discriminate active Lyme borreliosis from previous exposure”

Dear Sir,

Thank you for considering to publish this study and for providing valuable comments and suggestions with the assistance of the reviewers in order to improve the quality of the manuscript. Here follows the rebuttal letter with point-by-point responses to each raised question/comment. Should any further concerns arise, do not hesitate to let us know.

Best regards,

Ivar Tjernberg, MD PhD Associate professor

Journal Requirements:

#1. We believe that our manuscript meets the PLOS ONE style requirements.

#2. The phrase “data not shown” has been deleted from the manuscript at the end of the Results section. The important results and confirming p-values are shown in the written text stating that age and sex did not impact the odds ratio of the index.

Responses to reviewer comments

All changes made to the manuscript have been highlighted with yellow in the revised version in order to easily be detected.

**Reviewer #1**

Thank you very much for valuable comments and suggestions in order to improve the quality of this work and manuscript. We have revised the manuscript accordingly to the best of our ability, and here follows point-by-point replies to raised concerns:

The rationale for selecting the predefined immune response panel from Olink® has been added to the end of the introduction section.

The information regarding the blood donors self-reported frequency of previous Lyme borreliosis has been added to Table 1. In the revised Table 1 only those blood donors with a complete set of biomarker results are included (n=75), thus 5 blood donors were excluded. These 75 blood donors also correspond to the results presented in Table 2, Fig 1 and Supplementary Table 1, making comparisons more easily across tables and figure. This has been clarified in the beginning of the Results section.

As suggested, and in addition, we have also investigated the diagnostic strength of the index comparing the patients with active LB vs the subgroup of blood donors with *Borrelia*-antibodies and found an almost identical AUC in the ROC- analysis (0.973). The AUC comparing patients with all blood donors was 0.964 as shown in Fig 1. A sentence describing this sub-analysis has been added towards the end of the Results section. Thus, the discriminating strength of the index is as good regardless of prevalence of anti-*Borrelia* antibodies from a previous exposure.

Comments directly in the manuscript:

#1-3 Abstract. Both sentences under Objectives have been changed in accordance with the suggestions as well as the word “significant” changed to “significantly” under Results.

#4 Introduction. The sentence on row 104 has been changed as suggested.

#5 Data analysis and statistics. The wording regarding LOD has been changed according to the table, i.e. the number (%) of samples below LOD but not each specific LOD of the included parameters.

#6 Data analysis and statistics. Comment regarding the statistical analysis. The ten proteins were included in the regression analysis, not age and sex. However, age and sex were subsequently found to not impact the odds ratio of the six protein index. A sentence on this matter has been added to the end of the data analysis and statistics section. Regarding the question on cut off for the Mann-Whitney test at p=0.1 it was a carefully determined limit in order to balance the chance of discovering important biomarkers for active LB with the risk of obtaining irrelevant results due to statistical reasons. However, the approach of performing the tests on two halves of the study objects and then only further considering parameters that were simultaneously significantly different between the two study groups in both halves should counteract the risk of finding differences by chance alone. Furthermore, all individual p-values are accounted for in Table 2, and as shown, two of the initial parameters with p-values between 0.05-0.1 (MW) still remained in the final six biomarker regression index with p-values <0.05. So, the number of parameters included in the regression model seems to be adequate, and the markers still significant after the regression were also tested by the index in the ROC-analysis with a confident result. However, as this is a first and screening study, the findings certainly need confirmation, something that we also acknowledge in the Discussion. Finally, we also want to state that all statistical analyses in this study were planned and performed by an experienced statistician, also co-author for the manuscript, prof Lars Brudin.

#7 Discussion. The wording was changed according to the suggestion from “previous LB” to “previous infection” in row 243 of the original manuscript.

**Reviewer #2**

Thank you very much for reviewing our manuscript and providing valuable comments in order to improve the quality. We have tried to the best of our ability to clarify the manuscript accordingly and also to explain and give answers to raised questions.

Major concerns

Study design:

The study group with ongoing/active Lyme borreliosis (LB) patients (n=52) were all under investigation for ongoing complaints and according to two separate and independent physicians with great experience of Lyme borreliosis defined as patients with ongoing LB manifestations according to European case definitions (definite Lyme neuroborreliosis fulfilling all three criteria, Lyme arthritis and/or Acrodermatitis chronica atrophicans with clinical signs compatible with these manifestations confirmed with detection of *Borrelia*-specific DNA in synovial fluid/skin biopsy. Finally patients with typical erythema migrans skin rash >5 cm in diameter after recent tick-bite ). These LB patients were all sampled with ongoing symptoms/complaints and before antibiotic treatment initiation. Yes, indeed, these LB patients may also have had a previous episode of LB, information that is not known, but they are in any case still defined as ongoing infection due to ongoing complaints and diagnostic testing as described above. The blood donors, on the other hand, stated that they were all healthy at the time of sampling which was at a routine visit for blood donation, thereby with as much certainty as possible not diseased at the time of sampling. Still, the possibility of an asymptomatic or subclinical infection amongst the blood donors at sampling cannot be totally ruled out, but is considered highly unlikely. Therefore, the proportion of these blood donors with a positive anti-*Borrelia* antibody result is considered to have had a previous asymptomatic/symptomatic (now not active) encounter with *Borrelia*.

We have tried to clarify the section on study participants in the Material and methods section of the manuscript, please see changes highlighted in yellow in the revised manuscript. We believe that the individuals in the study were carefully defined and that they correspond well to the study purpose and thereby that the design of the study is valid.

Methodology:

It is true that false positive reactions may occur in *Borrelia* serological tests, most commonly regarding IgM. In this study, we therefore focused on the inclusion of blood donors with positive IgG-antibodies to reduce the risk of false positive results, and also primarily to detect individuals with a previous exposure to *Borrelia*. The test used for this was the commercial test Enzygnost Borrelia Lyme IgM/IgG (Siemens/DADE Behring, Marburg, Germany). Although, adsorption with E. coli was not performed, positive Enzygnost Borrelia antibody results were also confirmed in the independent *Borrelia* serological assay Liaison IgG and/or the C6 ELISA using variants and other *Borrelia* antigens.

One sentence regarding this has been added to the “Lyme borreliosis patient and blood donors” section under “Material and methods”. In general, we have tried to clarify the definitions of the study individuals and their respective clinical and laboratory status as far as possible under the Material and methods section. Additional information concerning the study material may also be found in the publication by Lager et al 2019, also referenced in the manuscript.

Minor comments:

Abstract:

Line 58. We have changed the wording from “Lyme borreliosis infection” to “Lyme borreliosis” since the term Lyme borreliosis already implicates infection. Also note additional changes in the wording highlighted in yellow in order to clarify. The cohort of LB patients were all sampled with ongoing=active infection as reflected by ongoing symptoms and complaints confirmed according to diagnostic criteria for various manifestations of LB. On the other hand all blood donors were healthy at sampling, thus a control group to compare the LB patients with, a proportion of whom were anti-*Borrelia* antibody positive, thus reflecting a previous *Borrelia* exposure. The information on the selection and definitions of the study subjects has been expanded and clarified in the Material and methods section. The word count for the abstract is limited and does not allow for further detailed description of the study groups.

Line 65. The wording “immune-related proteins” has been changed to “proteins”.

Line 68. The abbreviations have been removed from the abstract.

Methods:

Line 112, 116-117, 118-120. Much of the Material and methods section has been rewritten and clarified according to the raised questions and comments.

Line 120-122. Details on the Enzygnost kit and confirmatory kits have been added to the Material and methods section.

Line 125. Before each routine blood donation, each blood donors must complete a standardised health declaration including a number of questions regarding the current and recent health situation. The questions cover many aspects of health including a general statement of health and well-being, fever, recent symptoms of infection, other healthcare contacts including potential examinations or investigations, treatments and medications etc. A sentence has been added regarding this to the description of blood donors.

Line 130-141. This section describes the principles of proximity extension assay, i.e., a multiplex method that allows simultaneous detection and quantification of multiple human proteins in a single sample. The technique was first developed by Olink® in Uppsala, Sweden, later adopted by SciLifeLab at Uppsala University. A detailed description of the method and technique has been published by Assarsson et al 2014, now properly referenced in the section on the Laboratory analyses under Material and methods. The referenced paper provides a detailed explanation, and since 2014 some 600 of studies using the PEA method have been published according to PubMed database, making it an established method. Thus, we only briefly describe the method principle. To clarify, the method detects multiple human protein biomarkers in the investigated study subjects, not borrelial proteins. Some limited changes to this section of the manuscript have been applied.

**Reviewer #3**

Thank you for constructive comments on our manuscript. We greatly appreciate these valuable questions and opinions for us to improve the quality of the report. Here follows point-by-point replies to raised concerns.

Indeed, this paper is a first attempt at this approach, and although with promising findings, further confirmation is required – something we agree on and conclude in the end of the discussion.

#1. In the discussion, pages 18-19, we have tried to elucidate what is known of these six proteins. There is no previous report on any of them in the Lyme borreliosis (LB) field according to a PubMed search. Our understanding is that each of these biomarkers by itself is not specific for LB or any particular disease. Rather the specific combination of them presented as the index suggests a significant pattern enabling identification of active/ongoing LB. Additional studies may reveal if this response pattern is specific for LB or also may occur in other (active) infections. We have expanded on one sentence towards the end of the discussion including the possibility that this response pattern may also occur in other infectious and inflammatory conditions that need investigation.

#2. We have included a sub-analysis of the ROC-analysis on the discriminating power of the protein index based on the LB patients vs only those blood donors with a positive anti-*Borrelia* antibody test result and found an almost identical area under the curve indicating that the blood donor group may be handled as one. A sentence clarifying this has been added at the end of the Material and methods section.

#3. We have changed the title of the manuscript accordingly. It now reads: “Identification of potential biomarkers in active Lyme borreliosis”

#4. As requested, we have also performed an additional ROC curve analyses comparing Lyme neuroborreliosis patients (n=41) with blood donors (n=75). The resulting AUC of this sub-analysis (0.962) was not statistically different from the original AUC including all patients and blood donors (0.964). Please see additional information on these analyses at the end of the Results section.

#5. The baseline results for all 92 investigated protein biomarkers for both study groups are presented in supplementary table 1. In table 2, however, only those ten biomarkers that showed statistical difference comparing the groups and the following results of the logistic regression are presented. Thus, the content of S1 Table is not the same as Table 2.

Minor comment

Line 172. The sentence has been corrected.
